# Association of Edmonton Symptom Assessment System Global Distress Score With Overall Survival in Patients With Advanced Cancer

**DOI:** 10.1001/jamanetworkopen.2021.17295

**Published:** 2021-07-16

**Authors:** Ishwaria M. Subbiah, Maira M. Charone, Jason Roszik, Ali Haider, Marieberta Vidal, Angelique Wong, Eduardo Bruera

**Affiliations:** 1 Division of Cancer Medicine, Department of Palliative, Rehabilitation and Integrative Medicine, The University of Texas MD Anderson Cancer Center, Houston; 2 Division of Cancer Medicine, Department of Melanoma Medical Oncology, The University of Texas MD Anderson Cancer Center, Houston

## Abstract

This cohort study examines the association of the Edmonton Symptom Assessment, including the Global Distress Score, with overall survival rates in patients with advanced cancer.

## Introduction

**Figure 1.**
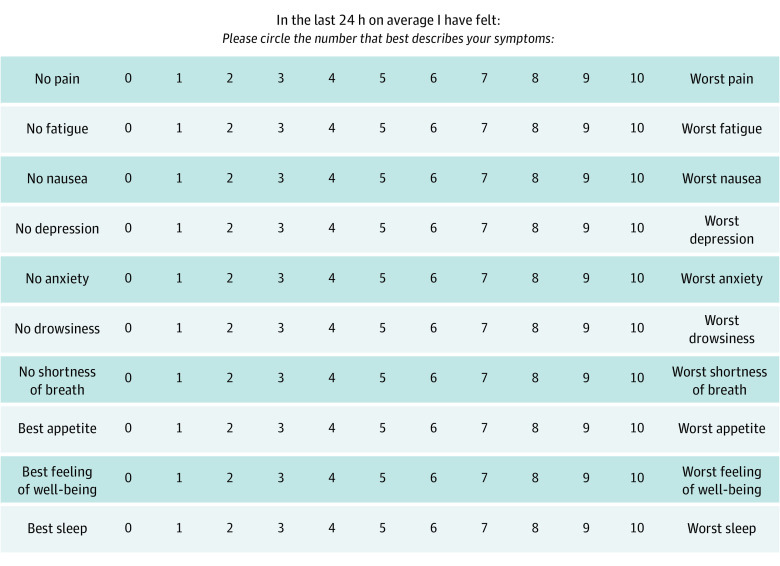
The Edmonton Symptom Assessment System (ESAS)

Despite compelling data supporting their use, patient reported outcomes (PROs) are not widely integrated into routine cancer care.^1^ Given the landscape of PRO instruments with convoluted scoring methods, we reflected on our own PRO-driven palliative care (PC) practice, where over the past 20 years all patients completed the Edmonton Symptom Assessment System (ESAS) at every visit. As a simple, validated 10-item PRO tool, the ESAS measures the following 10 common symptoms in advanced illness: pain, fatigue, nausea, drowsiness, appetite, dyspnea, well-being, anxiety, depression, and sleep (Figure 1).^2^ Validated subscales of the ESAS include the Global Distress Score (GDS), a sum of the first 9 physical and psychosocial ESAS symptoms.^3,4^ In this study, we examined the association between the ESAS and overall survival (OS) in patients with advanced cancers.

## Methods

In this cohort study, we queried a prospective database and identified consecutive patients with metastatic cancer with a first outpatient PC visit from January 1, 2018, to June 30, 2018. Prior treatments included systemic therapy, radiotherapy, and surgical procedures. This study received MD Anderson institutional review board approval with waiver of informed consent given that it was not interventional and captured data obtained as a part of routine clinical care. We followed the Strengthening the Reporting of Observational Studies in Epidemiology (STROBE) reporting guideline.

All patients completed the ESAS reporting their average symptom severity over the previous 24 hours on a scale of 0 (not present) to 10 (most severe). The GDS was calculated and grouped into 3 cohorts based on previous work and clinical experience as follows: high (GDS ≥35), moderate (16-34), or low (0-15). Overall survival was defined as time from the first PC visit date to death. Logistic regression analysis, analysis of variance, and *t* tests were conducted using IBM SPSS Statistics for Windows, version 24 (IBM Corp), and significance was set at 2-sided *P* < .05.

## Results

**Figure 2.**
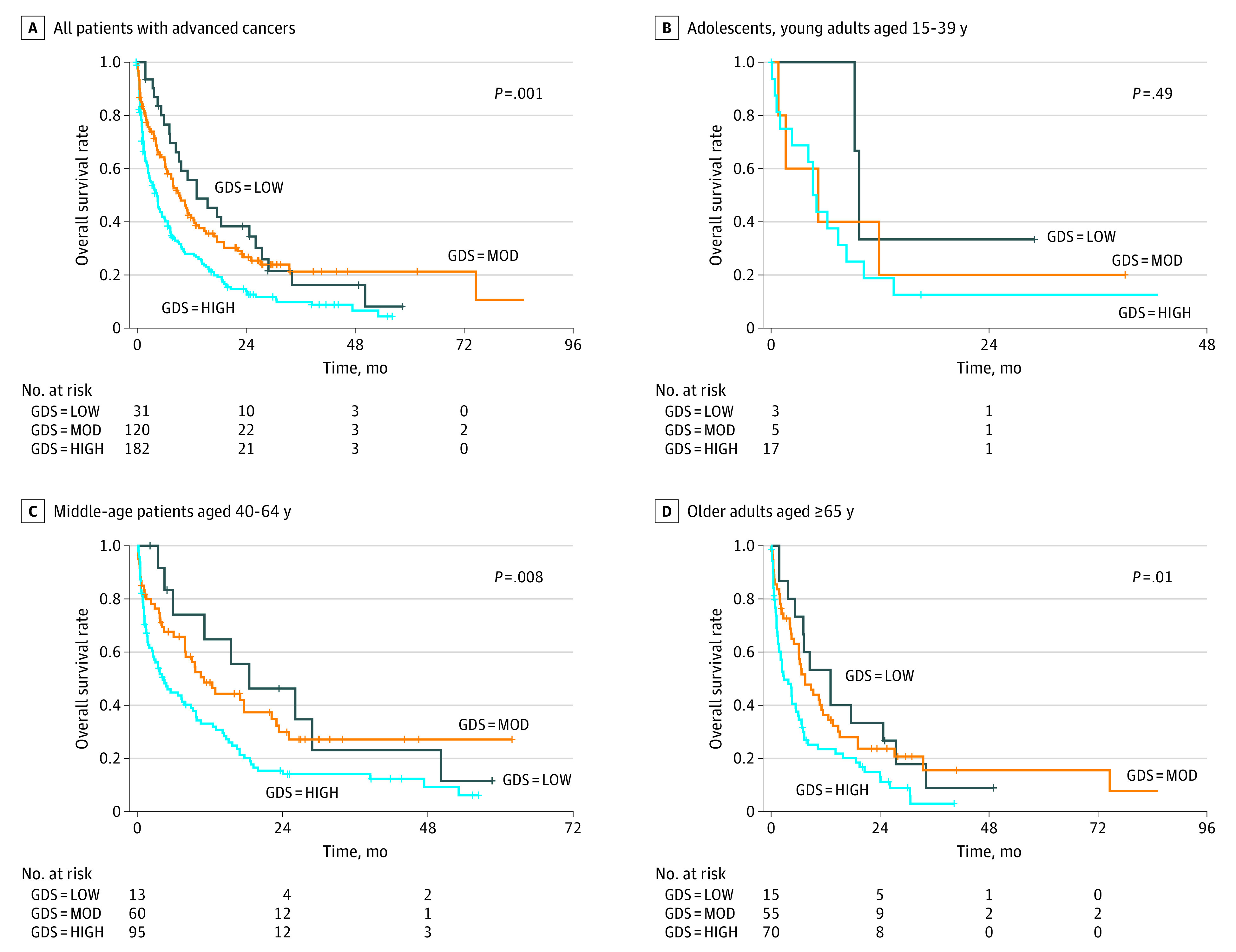
Overall Survival by Low, Moderate (MOD), and High Global Distress Score (GDS) Among Patients With Advanced Cancers

Among 333 patients with advanced cancer (190 women, 143 men) in the study, the median age was 62.4 years (range, 20.5-88.4 years) including 25 (7.5%) adolescents and young adults (AYAs) (aged 15-39 years), 168 (50.5%) middle-aged adults (40-64 years), and 140 (42.0%) older adults (≥65 years). The median number of prior treatments was 2 (range, 0-11); 227 of 333 patients (68.2%) were receiving second-line treatment and beyond. The median Eastern Cooperative Oncology Group (ECOG) Performance Status (PS) was 2 (ambulatory) (range, 0-4); 124 of 333 patients (37.2%) reported having a PS 3 (limited self-care) and 33 (9.9%) a PS 4 (completely disabled). At the time of analysis, 262 patients had died. Overall, a higher ECOG PS was also associated with higher GDS in this patient cohort. For example, the mean GDS of patients with PS 0 was 25.0 (IQR, 25-45) vs 38.5 for patients with PS 4 (IQR, 28-45) (*P* = .03). No significant differences in OS were observed between the 3 age cohorts (AYAs, 5.2 months; middle aged adults, 6 months; older adults, 5.4 months; *P* = .56). Overall, higher GDS was associated with lower OS (*r* = 0.21, *P* < .001), with an OS in the low GDS cohort of 13.1 months; moderate GDS cohort, 7.9 months; and high GDS cohort, 3.7 months (*P* < .001; Figure 2A). This association persisted within middle-aged adults (OS in the low GDS cohort, 15.5 months; moderate GDS cohort, 8.9 months; and high GDS cohort, 3.7 months; *P* = .008; Figure 2C) and older adults (OS in the low GDS cohort, 13.1 months; moderate GDS cohort, 6.7 months; and high GDS cohort, 2.6 months; *P* = .01; Figure 2D), but not in AYAs (OS in the low GDS cohort, 9.7 months; moderate GDS cohort, 5.2 months; and high GDS cohort, 4.6 months; *P* = .49; Figure 2B), likely attributable to the small AYA sample size.

## Discussion

The findings of this cohort study suggest that a higher GDS score was associated with a clinically significant decrease in OS highlighting the potential of the ESAS as a PRO tool in prognostication and clinical decision-making for patients with advanced cancers with a high symptom burden. While a higher ECOG PS was associated with having a higher GDS, obtaining the ESAS presents an opportunity for the clinical team to intervene on specific symptoms that contribute to an individual patient’s symptom burden and suffering. Limitations of this work include that it was a single-institution study in which PC referral was at the primary clinician’s discretion; consequently, patients of varying primary cancers and symptom burden are represented. In the realm of increasingly complex PRO instruments, the ESAS represents a simple validated tool that is completed in less than a minute, with subscales that are associated with OS among patients with advanced cancers.^5^
